# Medicine and the Individual

**Published:** 1919-03-22

**Authors:** 


The Hospital

The Workers' Newspaper of Administrative Medicine and Institutional Life, Administration, National Insurance and Health.

No. 1711, Vol. LXV. SATURDAY, MARCH 22, 1919. PRICE TWOPENCE

### MEDICINE AND THE INDIVIDUAL.

Under the pressure of certain social and international events there is at the present hour an almost inevitable tendency to think of the human race in collective rather than in individual terms. It is strictly and literally true that the fate of nations is trembling in the balance, and whole groups and classes of men and women are affirming a corporate status and urging a corporate demand. In such circumstances it is no matter for surprise that the individual unit seems of slight importance and escapes any considerable attention. Projects of national advance and development and of social readjustment and improvement are presented in attractive and confident tones, and it is obvious that the authors of these schemes are possessed by the conviction that what is good for the community must needs be pleasing to the individual citizen. Not only is there to be social advance and peace, but also personal happiness and content. There is, of course, every reason why all efforts which make for social justice and stability should be encouraged, and there is a quite sufficient justification for them in the plea that they serve the general welfare. History, however, teaches us, and this by innumerable examples, that many so-called reforms have proved in practice much less beneficial than their authors expected them to be, and probably after every instance of this order there could be found at least a minority ready to affirm that the reform had done harm rather than good. Such a reflection presents a sad aspect to the enthusiast, and might perhaps advise him to be careful not to overstate his case. It is fortunate, on the whole, that such teachings from the past exercise little practical influence on those who feel they have a present-day crusade to preach. Disappointments in the past there may have been, but no such result is possible in the instance now in question. The outlook is sure and universal salvation quite beyond doubt. It is under the influence of such convictions that the energy necessary for advance is secured, and though the older generation listens with some incredulity there is ever a younger one ready to knit deeds to words. And both may perhaps agree that even a partial success is better than content with stagnation. Let such a formula be fully granted, and let a general benefit be recognised, it will still remain true that in every instance there will be individuals to be considered, and not all of these will join in the chorus of approval.

Perhaps more than most men the medical practitioner has his attention fixed on the unit rather than on the race, and he is conscious in a special measure that personality has in all schemes and plans to be taken into consideration. His first duty and care are with the individual patient, and to this interest he has to bend his thought and ambition. Not only so, but in the discharge of this responsibility he is continually impressed with the wide and commanding influence of personality, and with differences real enough in all conscience yet incapable of scientific classification and division. It was to meet such a position that physicians and philosophers of an earlier day elaborated a scheme of "temperaments," and found such labels as sanguine, melancholic, bilious, and the rest as appropriate tickets for particular classes. But even if it be allowed that there is a general basis of truth in the scheme, its application continually fails when confronted with individual cases. There are broad differences between "temperaments," as there are broad differences between nations, but the statement has a general not a particular value. Nor do the more exact experiences included in medical training yield a more helpful result. Indeed, they are less helpful, for they would say that both in structure and function man in the last analysis is identical with his neighbours. To the zoologist or the anatomist or the physiologist or the chemist, Brown is the same as Jones and Jones does not differ from Robinson. All three have the same essential elements arranged in the same essential fashion, and whether judged by morphological characters or by embryological development each one of the trio may be confidently and triumphantly placed with his fellows in one and the same generic classification. Upon such a basis it would seem fair to reason that what is good for one man will be good for all, and that what suits the community must needs be pleasing to its individual members. As we have said already, this assumption is tacitly accepted by not a few ardent advocates of particular social and international enterprises, and to throw doubts on its validity would in some quarters produce much surprise. Yet such doubts rest on a basis of exact observation, and no one is more conscious of them than the medical practitioner. The truth is that while men have certain characters in common each has his own individual features and capacities for response, and these, while they have their inconvenience for the legislator, are not without personal and social values. To the doctor some of these features present a special demand for estimate and recognition when he is called upon to offer advice in reference either to the prevention or to the cure of disease. They impress upon him some distrust of proposals which, handling mankind in the mass, announce a new heaven and a new earth without exception or qualification. And they certainly suggest that whatever new schemes be needed to fit the advance of habit and of thought, such schemes will be well-advised to avoid a rigidity which would repress the capacity for personal assertion and development. We all want the world to be "more and more." But such an end will not be obtained by the "withering" of the individual.

